# Assessing the environmental impacts of the Capilla del Monte wildfire in Punilla Valley of Argentina using Landsat-9 and Sentinel-5P

**DOI:** 10.1007/s10661-025-14374-y

**Published:** 2025-07-23

**Authors:** Furkan Yilgan, Nilay Yildiz, Tugba Dogan

**Affiliations:** 1 Department of Soil Science and Soil Protection, Faculty of Agrobiology, Food and Natural Resources, Czech University of Life Sciences Prague, Prague, Czech Republic; 2 Graduate School, Ondokuz Mayis University, Samsun, Turkey; 3 Faculty of Environmental Sciences, Czech University of Life Sciences Prague, Prague, Czech Republic

**Keywords:** Air quality, Google Earth Engine, Natural hazards, Punilla Valley, Remote sensing, Wildfire

## Abstract

Wildfires are a growing environmental concern due to rapid population growth, urbanization, and human activities, which contribute to climate change, causing wildfires that damage ecosystems and the environment. Wildfires destroy the vegetation cover, habitat of habitants and cause soil deterioration by changing the soil structure. In addition, toxic gases released into the atmosphere during fires threaten the lives of habitants. The effects of the forest fire that occurred on 19th September 2024 around Capilla del Monte in the Punilla Valley were analyzed using spectral indices. Landsat-9 data were used to detect changes in vegetation cover, land surface temperature (LST), and soil moisture by comparing the pre- and post-fire satellite images, while Sentinel-5P TROPOMI satellite data were used to extract the concentration of nitrogen dioxide (NO_2_) and carbon monoxide (CO) gases. Overall accuracy of the LST was found using a reference data MODIS daily LST, and a positive correlation (*r* = 0.94) found between the two datasets. In addition, the burned areas were estimated using the dNBR index as well as random forest (RF) and support vector machine (SVM) classification methods. The results showed that vegetation cover increased by 35%, the average soil moisture decreased by approximately 16%, and the average LST increased by 9.5% from October 2023 to November 2024 in the region. The burned area was estimated as 387.9 km^2^ using dNBR, while it was 392.4 km^2^ by RF and 389.5 km^2^ by SVM in the study area. The study found high NO_2_ and CO concentrations after the Punilla Valley fire, threatening inhabitants.

## Introduction

Wildfires are widespread phenomena due to high temperatures and global warming on the Earth (Shi & Touge, 2022). Although wildfires occur from natural causes, many fires are the result of human activity, including accidents or intentional actions (Grala et al., 2017; Kolanek et al., 2021). Wildfires cause huge damages in the environment by affecting life of habitants, especially in forest ecosystems (Kelly & Peng, 2024). Additionally, they also disrupt soil structure, which harms soil-dwelling organisms and hinders future plant growth (Farid et al., 2024). Moreover, wildfires cause the organic matter and aggregate stability of the soil to be affected and reduced, which paves the way for soil erosion (Agbeshie et al., 2022). They can also lead to forest destruction and influence vegetation regrowth, both of which can affect water resource availability by altering evapotranspiration (Meili et al., 2024). In addition, soil evaporation increases due to wildfires, while soil infiltration decreases because of fine ash particulates resulting from the burning of organic matter in the soil. Thus, groundwater hydrochemistry can be affected by ash residues that may seep into the soil and affect groundwater quality (Rodríguez-Jiménez et al., 2024). On the other hand, wildfires that cannot be controlled spread over large areas and reach towns and settlements, causing great damage to people ‘s homes and infrastructure (Shi et al., 2021). This situation causes great economic losses in the countries where it occurs, and wildfires pose a major threat to people and property (Argañaraz et al., 2017). Moreover, various poisonous gases and aerosols released because of fire and spread into the atmosphere also threaten the lives of habitants (D’Evelyn et al., 2022). Particularly, nitrogen dioxide (NO_2_), carbon monoxide (CO), methane (CH_4_), and sulphur dioxide (SO_2_) gases can be given as examples (Halder et al., 2023). Apart from this, the layer formed by these poisonous gases spreading into the atmosphere prevents solar radiation from reaching the Earth (Odubo & Kosoe, 2024). High temperature caused by the burning of organic matter produces NO_2_, whereas incomplete combustion of carbon-containing materials like wood and grass produces CO (Wan et al., 2023). While CO binds to blood hemoglobin, decreasing oxygen delivery and causing vertigo, NO_2_ can increase respiratory tract inflammation and illnesses like asthma and bronchitis (Khajeamiri et al., 2021).

Remote sensing (RS) is widely used because of fast monitoring and early detection opportunity of environmental hazards, although ground data is one of the techniques for the investigation of wildfires and air quality assessments (LoPresti et al., 2024; Park et al., 2024). Thus, some spectral RS indices such as normalized difference vegetation index (NDVI), normalized burns ratio index (NBR), normalized difference moisture index (NDMI), soil moisture index (SMI), and land surface temperature (LST) are used for damage detection due to wildfires as well as creating fire risk assessment maps (Sivrikaya et al., 2024). According to a study by Widya et al. (2024), changes in NDVI, NBR, NDMI, and soil-adjusted vegetation index (SAVI) were calculated using Sentinel-2 RS data to assess wildfire severity and vegetation cover changes in Gangwon Province, South Korea. Wildfire severity and changes in vegetation cover were successfully detected, and statistical analyses revealed significant correlations between fire severity levels and environmental impacts. The lowest correlation on the changes between indices was *r* = 0.62, while the highest was *r* = 0.98. In another study, Yilgan et al. (2025) investigated a forest fire that occurred in a popular tourist area in Czechia using spectral RS indices derived from Landsat-8 satellite data. NDMI, SMI, and LST were compared before and after the wildfire. The study demonstrated pre- and post-fire change maps of the Bohemian Switzerland National Park and estimated the burned area using NDVI and NBR indices. Moreover, Xu et al. (2024) calculated NDVI and NBR indices in northeastern Alberta, Canada, to detect burn severity caused by wildfire. Their study found that 53.51% of the affected area experienced burns of either low or high intensity. Furthermore, Liu et al. (2023) calculated NBR and NDMI indices from RS satellite data following bushfires in western South Australia. Their results demonstrated that while NDMI is a good predictor of post-fire recovery, it is less effective at detecting fire events in arid regions due to low signal amplitudes. Additionally, Chernysh and Stakh (2024) calculated NDVI, NBR, and their difference to detect burned areas due to oil pipeline explosions near Strymba Village, Ukraine. They also analyzed the distribution of CO and NO_2_ gases following the wildfire using Sentinel-5P RS satellite data. The study demonstrated that NDVI and NBR are effective indicators for estimating burned areas. Moreover, Mashhadi and Alganci (2021) estimated burn severity using Landsat-8 and Sentinel-2 RS data by calculating the differenced normalized burn ratio (dNBR), difference normalized difference vegetation index (dNDVI), and applying support vector machine (SVM) and random forest (RF) machine learning (ML) classification methods. The study showed that dNBR provided more accurate results than dNDVI, and both ML supervised classifications were reliable predictors. Furthermore, Magro et al. (2021) used Sentinel-5P TROPOMI RS data to calculate CH₄ and CO concentrations to assess changes in air quality following a wildfire in Portugal. The study results showed a clear trend and pattern between CO and in situ measurement indicating that CO obtained from Sentinel 5P is reliable.

RF and SVM are widely used supervised ML algorithms for burned area estimation because of their ability to model non-linear relationships in multispectral data and reduce the risk of overfitting in high-dimensional feature spaces (Sayad et al., 2019). In addition, these methods are particularly effective in classifying satellite-derived indices (e.g., NDVI, NDMI, NBR) for post-fire assessments. Chen et al. (2024) compared RF, SVM, and neural networks (NN) in detecting fire-affected areas in Xintian County, China, using Sentinel-1B and 2A data. Their results revealed that SVM provided the highest accuracy in the pre- (93.52%) and post-fire (94.97%) periods when combined with spectral, NDVI, or radar features, while RF achieved the best performance (95.43%) during the fire event when using spectral features with the NBR index.

Argentina experienced many forest fires throughout its history, and many of the wildfires were understood to be caused by human impacts. The Punilla Valley has been significantly affected by wildfires during the 2023 and 2024 wildfire season in the Córdoba Province in Argentina, though. The forest fire started on 19th of September 2024 near Capilla del Monte, a small town in the Punilla Valley near Córdoba city in Argentina. Authorities have deployed a large number of firefighters and fire trucks to the area to prevent the fires from reaching residential areas. Fire affected not only human life and other habitants of forest ecosystems negatively, but also the infrastructure and houses. According to the news by Iricibar (2024), it was required to evacuate 54 people due to the destruction of more than 20 homes in the Punilla Valley on 23rd of September. In addition, three people were also arrested for intentionally starting the wildfire. Furthermore, the Punilla Valley had another a serious human-caused wildfire in October 2023, near the city of Villa Carlos Paz. Hundreds of people were evacuated from their homes, and approximately 1000 firefighters, eight firefighting planes, and two helicopters responded to the fires (Ross, 2023).

According to literature, many studies have been conducted on the effects of wildfires; there are not many studies that integrate many RS indices and conduct comprehensive analyses of parameters such as NDVI, NDMI, NBR, SMI, and LST. In addition, the newness of the Landsat-9 data series to be used with many spectral indices in the study makes methodology unique by analyzing the fire in the Punilla Valley. In addition to all of this information, since the Punilla Valley is a very important touristic area in Argentina, the protection of natural heritage areas in the region and the characterization of forest fires specific to the region are of great importance. The study aims to investigate changes in vegetation cover, moisture content of the soil and plants, as well as air quality changes using RS satellite data after the wildfire occurred in Punilla Valley of Córdoba Province in Argentina. The study findings will provide fast and reliable information about the destroyed areas in the valley and will also contribute to the future ground analysis to be carried out in the region. In addition, the study will shed light on both biodiversity research and ecosystem studies, as well as it may also contribute to forest lawmakers and the regulation of forest laws in vulnerable regions to wildfires. Spectral RS indices can provide quantitative, location-specific data on the impacts of wildfires, thus enabling policymakers to understand the scale and impact of wildfires and design targeted policies based on evidence. In this context, spectral RS indices such as NDVI, NBR, NDMI, LST, and SMI were calculated, and their statistical relationships were assets for the interpretation of changes after wildfire. The burned area was estimated using the difference of NBR indices, as well as RF and SVM supervised ML classifications pre and post fire.

## Materials and methods

### Study area

**Fig. 1:**
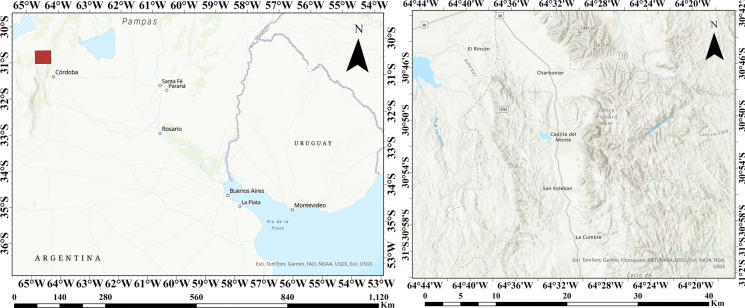
Country scale map of the region (left), total study area (right)

The study area (SA) is located at latitude 30° 54′ S and longitude 64° 32′ W, which covers Capilla del Monte, Los Cocos, Santa Sabina, Los Sauces, and San Esteban in the Punilla Valley of the Córdoba Province, Argentina. The total study area is shown on the right side in Fig. 1, and it covers 1955.17 km^2^ that has natural landscapes, rivers, lakes, and mountain villages. Adventure tourist activities such as hiking, horseback riding, rappelling, and water sports are popular in the Punilla Valley. Hosting occasions like the yearly National Folklore Festival in Cosquín causes it to have cultural value. Capilla del Monte is also a well-known small town with many touristic attractions in Argentina, and the town is located near the Sierras Chicas mountains with approximately 16,000 population (Papalini et al., 2022). In addition, the Punilla Valley has been influenced by tectonic and seismic activities associated with the geological evolution of the Sierras de Córdoba, which is a part of the Pampean Mountain Range (Dahlquist et al., 2024). The altitude of the valley varies between 500 and 1400 m above sea level, with an average of around 800 m. The climate of the Punilla Valley is mild and temperate, with some regional variations due to its elevation and geography (Suárez et al., 2022). Air temperature is between 5 and 15 °C during winter, while it is 15 and 25 °C in spring.

### Datasets

Landsat-9 Operational Land Imager (OLI) and Thermal Infrared Sensor (TIRS) RS optical satellite data were chosen due to the thermal observation opportunity of the satellite. Satellite images were chosen as pre- and post-fires that are on 29th October 2023, 12th August 2024, and 16th November 2024. The cloud coverage of RS images is less than 10%, and the spatial resolution of the Landsat data is 30 m. The spring season covers from September to December in the region, while August is the winter season. The selection of dataset dates was guided by the objective of comparing conditions immediately following the wildfire with those observed on the same date 1 year ago. Accordingly, October 2023 and November 2024 were selected for analysis. August was also included, as it was deemed valuable to examine the state of the region just before the wildfire, although it was falling within the winter season. Moreover, the rainfall data for Capilla del Monte were checked using the historical data archive from the meteorological website https://www.wunderground.com for the dates when the satellite images were taken. It was seen that there was no rainfall information in the region on all the dates. In addition, Sentinel-5P TROPOMI satellite images that have 7 km × 3.5 km spatial resolution were used to extract air quality data. The spectral ranges that Sentinel-5P monitors include ultraviolet (UV), visible, near-infrared (NIR), and shortwave infrared (SWIR). The Sentinel-5P datasets were chosen between 14th September–19th September for pre-fire and 21 st September–25th September after the wildfire started.

### Methodology

**Fig. 2:**
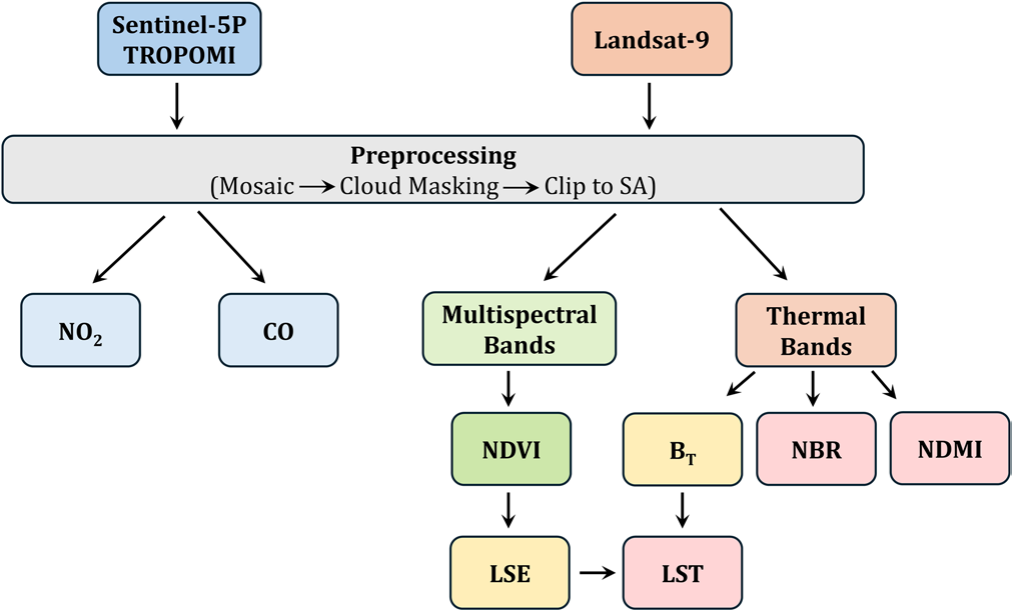
Flowchart of the methodology steps of spectral indices and gases

RS data were assessed using a cloud-based Google Earth Engine (GEE) platform, which has a huge amount of satellite dataset for environmental analysis (Gorelick et al., 2017). RS images were masked to remove cloud effects and clipped to the study area. The basic step of methodology is shown in Fig. 2 as a flowchart.

#### Calculation of normalized difference vegetation index

The normalized difference vegetation index (NDVI) is an indicator for discrimination of soil and vegetation cover, and values exceeding 0.2 generally represent areas covered with vegetation (Sashikkumar et al., 2017). The NDVI is computed using the red and near-infrared bands of the Landsat-9 dataset using the formula provided in Eq. (1) (Tucker, 1979).

1 $$NDVI = (NIR- R) / (NIR + R)$$

where *NIR* is the near-infrared band, and *R* represents the red band of Landsat-9 satellite, while *NDVI* is vegetation density as well as health, foliage, and phenology of plants in the study area.

#### Normalized burn ratio index (NBR)

The normalized burn ratio index (NBR) helps to estimate burned areas due to the difference between the reflectance of healthy vegetation and burned vegetation in the short-wave infrared interval of the electromagnetic spectrum. NBR is calculated using the short-wave infrared and near infrared bands of the satellite using the formula that is given in Eq. (2) (Smith et al., 2005):

2 $$NBR =(NIR-{SWIR}_{2})/\left(NIR + {SWIR}_{2}\right)$$

where *NIR* is the near-infrared band of the satellite, and *SWIR*_2_ is the short-wave infrared band of the satellite that has 2.11–2.29 µm wavelength, whereas *NBR* is the normalized burn ratio index. The NBR index is used to detect burned areas by differenced normalized burn ratio (dNBR). The dNBR was obtained by the difference of the pre-fire and the post-fire (October 2023 and November 2024) NBR values.

#### Normalized difference moisture index (NDMI)

Normalized difference moisture index (NDMI) represents moisture content of plants (Siachalou et al., 2009). NDMI is calculated using near infrared and shortwave infrared bands of Landsat-9 satellite images. The NDMI has a range between − 1 and 1, and it was calculated by a formula that is given in Eq. (3) (Jin & Sader, 2005).

3 $$NDMI =(NIR-{SWIR}_{1})/\left(NIR + {SWIR}_{1}\right)$$

where *NIR* represents the near-infrared band of Landsat-9 satellite, *SWIR*_1_ is the first shortwave infrared band of the satellite, and its wavelength is between 1.57 and 1.65 µm.

#### Derivation of land surface emissivity (ε)

Land surface emissivity (LSE) values are determined through the NDVI thresholding method, where values less than 0.2 represent bare soil, those exceeding 0.5 correspond to vegetation, and values between 0.2 and 0.5 denote mixed pixels (Sobrino et al., 2004). The emissivity of bare soil is equivalent to the red band’s reflectance values, vegetation-covered pixels possess an emissivity of 0.99, and emissivity for mixed pixels is calculated using the formula presented in Eq. (4) by Valor and Caselles (1996) and Sobrino and Raissouni (2000).

4 $$\varepsilon = \varepsilon V * {P}_{V} + \varepsilon S (1 - {P}_{V}) + d\varepsilon$$

where *ε* means emissivity, *εV* represents vegetation emissivity, *εS* denotes soil emissivity, *dε* is the geometry factor of the object, and *PV* denotes the vegetation proportion calculated using the minimum and maximum NDVI values. *P*_*V*_ is calculated using Eq. (5) with the minimum and maximum NDVI values set at 0.2 and 0.5, respectively.

5 $${P}_{V} = {((NDVI-{NDVI}_{\text{MIN}})/({NDVI}_{\text{MAX}}-{NDVI}_{\text{MIN}} ))}^{2}$$

The geometry factor of the object, *dε*, is calculated using Eq. (6), where *F* is a constant reflecting the Earth topography’s shape factor, typically estimated at 0.55 (Sobrino et al., 1990).

6 $$d\varepsilon = (1 - \varepsilon S) (1-{P}_{V}) F\times \varepsilon V$$

#### Determination of satellite brightness temperature (BT)

The satellite’s brightness temperature is derived through the conversion and calibration of raw satellite data, which is initially stored as digital numbers without any intrinsic unit. The conversion of raw satellite image pixels into spectral radiance (TOA) values is obtained using the formula provided in Eq. (7) (U.S. Geological Survey).

7 $${L}_{\lambda }={M}_{L}\times {Q}_{CAL}+ {A}_{L}$$

where *L*_*λ*_ represents the spectral radiance values of satellite images, the band of the raw data of the satellite is *Q*_*CAL*_, and *A*_*L*_ and *M*_*L*_ are factors that are stored in the metadata file of the satellite (U.S. Geological Survey). *M*_*L*_ serves as a rescale factor for the satellite, whereas *A*_*L*_ is an additive rescale factor. Subsequently, the brightness temperature values are calculated through the usage of thermal conversion constants of the satellite that are *K*_1_ and *K*_2_, as given in Eq. (8).

8 $${B}_{T} = {K}_{2} / (ln ({K}_{1} / {L}_{\lambda }) + 1)$$

*L*_*λ*_ signifies spectral radiance, and *B*_*T*_ shows the satellite’s brightness temperature in Kelvin (K) units. According to the Kelvin temperature scale based on thermodynamic experiments by Guildner and Edsinger, (1973), 273.15 K is equal to 0 Celsius (°C).

#### Computation of land surface temperature (LST)

Land surface temperature is calculated using the emissivity and satellite brightness temperature parameter as well as Planck’s constant. Emissivity is an important parameter for the accuracy of the LST. The LST’s calculation formula was described by Dash et al. (2002) given in Eq. (9).

9 $$LST = {B}_{T}/(1+(\lambda \times {B}_{T}/\rho ) ln(\varepsilon ))$$

Here,* B*_*T*_ represents the satellite’s brightness temperature in Kelvin unit, while *ε* is land surface emissivity, *λ* is the mean wavelength of emitted radiance value, and *ρ* represents the blackbody law constant that is commonly referred to as Planck’s constant.

#### Soil moisture index (SMI)

Soil moisture index is calculated using the difference of the highest and the lowest LST values, and the calculation formula of SMI is given in Eq. 10 (Parida et al., 2008):

10 $$SMI = ({LST}_{\text{Maximum}}- LST) / ({LST}_{\text{Maximum}}- {LST}_{\text{Minimum}})$$

where *SMI* is soil moisture index and *LST* represents the layer of surface temperature values. *LST*_Maximum_ is the highest value of LST, while *LST*_Minimum_ is the lowest value of LST in the study area.

#### Estimation of burned areas

Random forest (RF) and support vector machine (SVM) are ML supervised classification techniques that were used for estimation of burned areas. NDVI, NDMI, NBR, LST, and SMI indices were used as input data with training sets such as 1400 selected spatially homogeneous pixel samples, which are 700 unburned pixels and 700 burned pixels in the study area. Sampling pixels were not randomly selected, and they were selected stratified and distributed spatially homogeneously everywhere in the study area. Determination of burned and unburned pixels was done by visual inspection of the satellite imagery, without using any raster layer of spectral indices. This method prevents any data leakage or bias in the training data. All pixels were used to construct the training and validation dataset with a ratio of 70% and 30%, and burned and unburned areas were separated from each other. Burned areas were estimated in km^2^ using the pixel numbers covered by the classes.

#### Air quality analysis

Changes of air quality were investigated using Sentinel-5P TROPOMI satellite that was developed by the European Space Agency (ESA) and was launched on 13 October 2017. The NO_2_ and CO are not direct bands of Sentinel-5P, but rather products derived from the satellite’s spectral observations. These gases are estimated by ESA using advanced atmospheric retrieval algorithms applied to raw spectral measurements. Sentinel-5P’s TROPOMI instrument collects high-resolution spectra of Earth’s atmosphere, and since each gas absorbs light differently at specific wavelengths, the concentration of each gas can be determined through spectral analysis. The NO_2_ and CO gases were extracted from the Sentinel-5P dataset for 5 days before the fire and 5 days after the fire on GEE platform (Shabbir et al., 2024). Mean data of those 5 days were compared with each other to see changes of NO_2_ and CO gases.

#### Statistical validation

The accuracy assessment of the LST was done by using Moderate Resolution Instrument Spectrometer (MODIS) reference data for a better understanding of the reliability of the study results. MODIS provides daily LST data with a 1 km spatial resolution. Landsat-9 data were rescaled to the same spatial resolution with MODIS to obtain more accurate results from the accuracy assessment. The correlation coefficient (*r*) and the root mean square errors (RMSE) were calculated between Landsat LST and MODIS LST. The statistical analyses were done by using 927 spatially homogeneously distributed pixel points that are equally distributed everywhere in the study area. Fishnet grids that ensure equal distance between points were created for obtaining the spatially homogeneous pixel samples, ensuring distribution and minimizing spatial sampling bias. The selected points for correlation statistics were entirely independent from the training dataset that was used in the burned area classification. In addition, statistical relationships between spectral RS indices were found by using Statistica v13 (TIBCO Software Inc.).

## Results

**Fig. 3:**
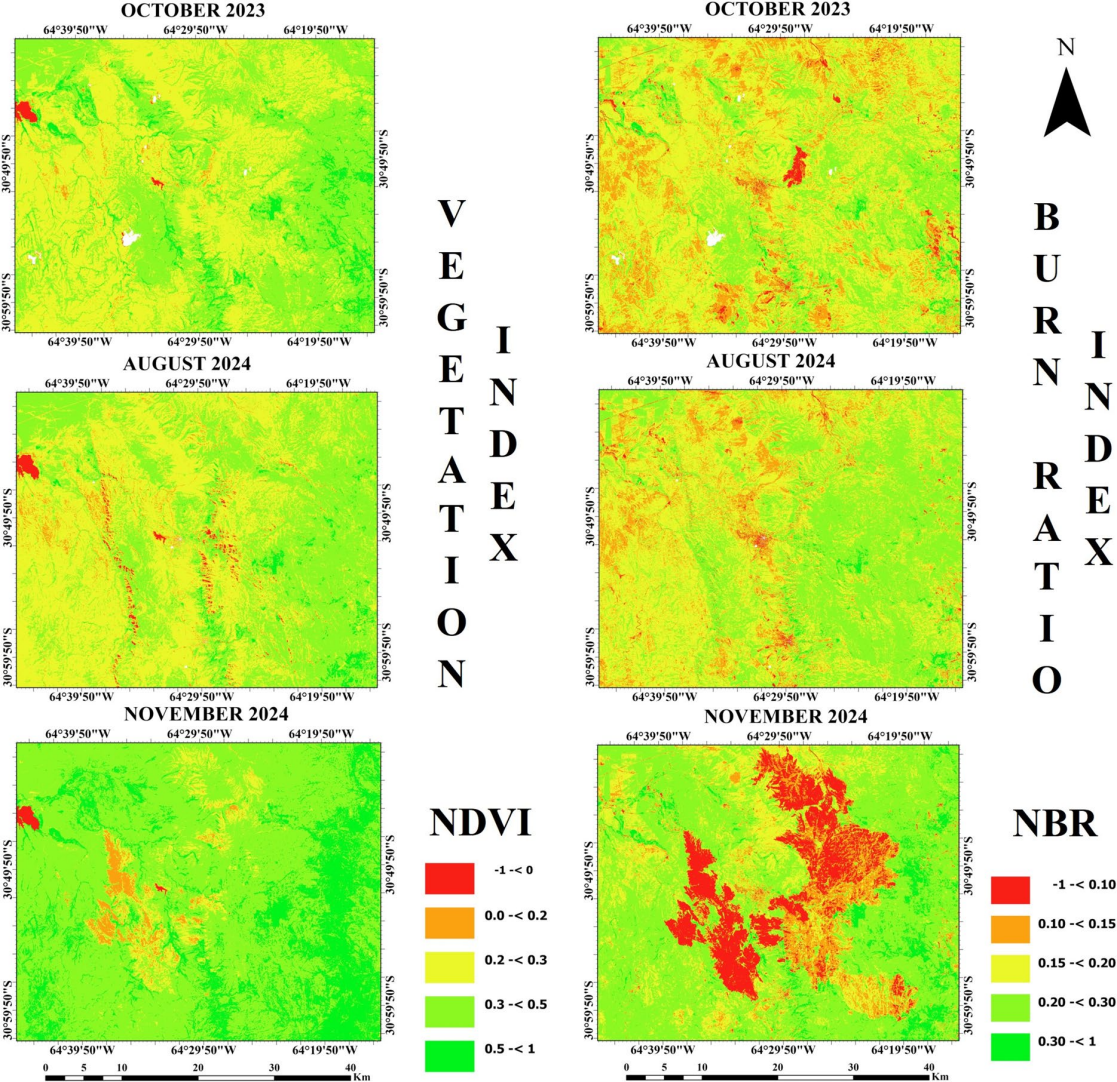
NDVI (left) and NBR (right) maps of the study area

NDVI, NBR, NDMI, LST, and SMI spectral RS indices were analyzed to detect changes in vegetation cover and moisture content of the soil and plants after the wildfire in the Punilla Valley in the Córdoba Province in Argentina. According to the results, the NDVI and NBR maps shown in Fig. 3 demonstrated that August 2024 does not show any significant change compared to October 2023 and does not have more vegetation. The red areas observed in August 2024 are due to the lack of vegetation because of the winter season on the top of some hills extending north–south and east–west of Capilla del Monte. In addition, the mean values of spectral indices, which are given in Table 1, show that the mean NDVI values were 0.32 and 0.30 in October 2023 and August 2024, respectively. NDVI and NBR values both have a range between − 1 and 1, and NDVI values lower than 0.2 represent non-vegetation. In addition to this, NBR values close to 1 indicate the presence of healthy vegetation, while values close to − 1 indicate burned areas (Tonbul et al., 2016). The vegetated areas are generally represented as green on the NDVI map in November 2024, while water bodies, bare soil, and the burned areas are represented by red and orange. Moreover, the areas shown in red color represent the water bodies and burned areas on the NBR map in November 2024. In addition, the NBR values in November 2024 decreased significantly and took values ranging mostly between − 1 and 0.1. Thus, burned areas can be observed by comparing November 2024 with October 2023, but it is important to remember that the values between 0 and 0.1 can also represent dry soil or brown burned vegetation. Although the NBR map from October 2023 represents the pre-fire period, a red area is visible near the center of the map. This area corresponds to the summit of Cerro Uritorco Mountain, which appears reddish due to its rocky structure. In the NDVI map from the same date, it also shows low values between 0 and 0.2, indicating bare soil.

**Table 1:** Mean values of spectral indices and their standard deviations (SD) over the years

Mean values	NDVI	SD	NBR	SD	NDMI	SD	LST(°C)	SD	SMI	SD
October 2023	0.32	0.09	0.18	0.04	− 0.13	0.10	25.6	3.25	0.37	0.07
August 2024	0,30	0.09	0.19	0.04	− 0.10	0.10	8.4	2.05	0.76	0.05
November 2024	0.43	0.11	0.20	0.07	− 0.02	0.12	28.0	3.05	0.31	0.07

**Fig. 4:**
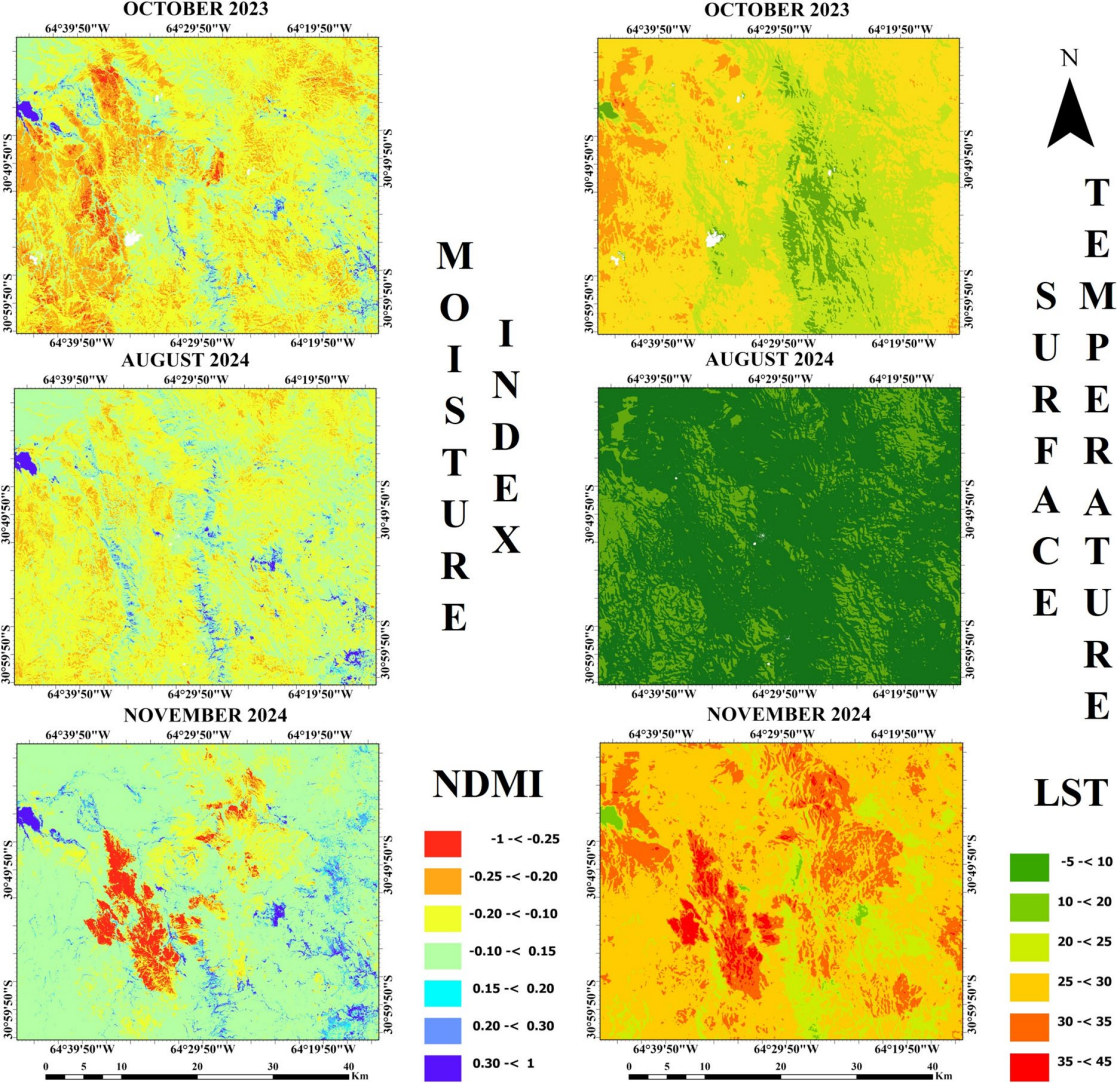
The NDMI (left) and LST (°C) (right) maps of the study area

The NDMI and LST maps shown in Fig. 4 demonstrate that the lowest LST values were in August 2024, while the LST had lower values in November 2024 compared with October 2023. The highest LST value was also found as 41.7 °C in November 2024, while the lowest value was found in August 2024 as − 2 °C. In addition, the lowest LST value was found at 5 °C in November 2023, but it was 15.6 °C in November 2024. Moreover, the highest NDMI value was found as 0.79 both in October 2023 and August 2024, while the lowest NDMI value was − 0.58 in August 2024. In addition, NDMI values approaching 1 indicate that the water content in plants is sufficient and moisture levels are high, while values approaching − 1 indicate low moisture and increasing water stress in plants (Raymond Hunt et al., 2012).

**Fig. 5:**
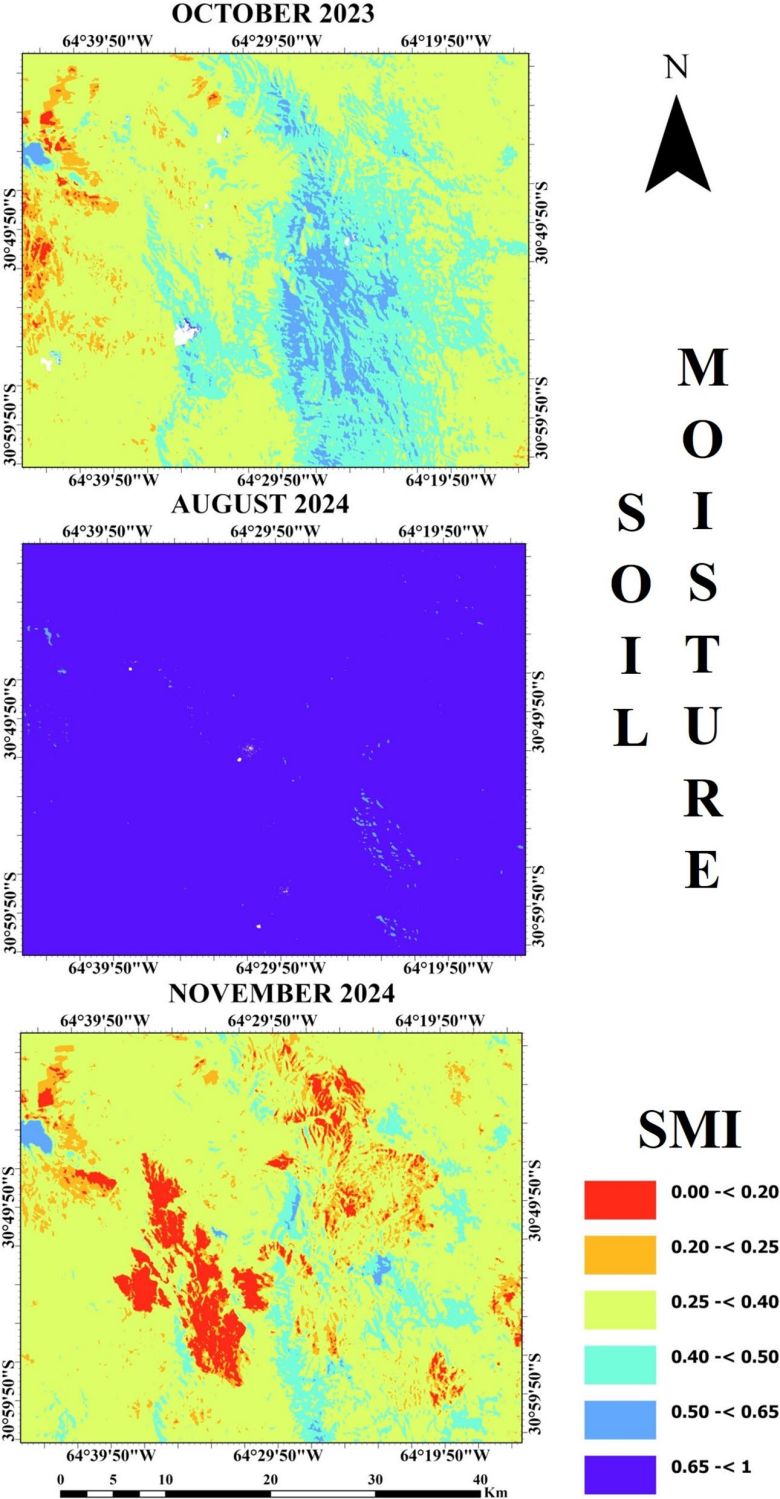
SMI maps of the study area

According to SMI maps that are shown in Fig. 5, the higher soil moisture values were observed in August 2024. The lowest SMI value was found close to 0 in November 2024, while the highest SMI value was in August 2024 near 1. Comparison of SMI map in November 2024 with SMI map in October 2023 shows that red color areas were observed more in November 2024. Thus, the SMI values appear to be lower in November 2024.

Mean values of spectral RS indices and their standard deviations (SD) are given in Table 1. The highest mean NDVI was found in November 2024 as 0.43, while the lowest mean value was 0.30 in August 2024. A small increase in mean NBR was found from 0.18 to 0.20 over time. Mean NDMI has an increasing trend from October 2023 with − 0.13 to November 2024 with − 0.02. The highest mean LST was found at 28 °C in November 2024, while the lowest was 8.4 °C in August 2024. The lowest SMI was 0.31 in November 2024, while the highest was found in August 2024 as 0.76.

**Fig. 6:**
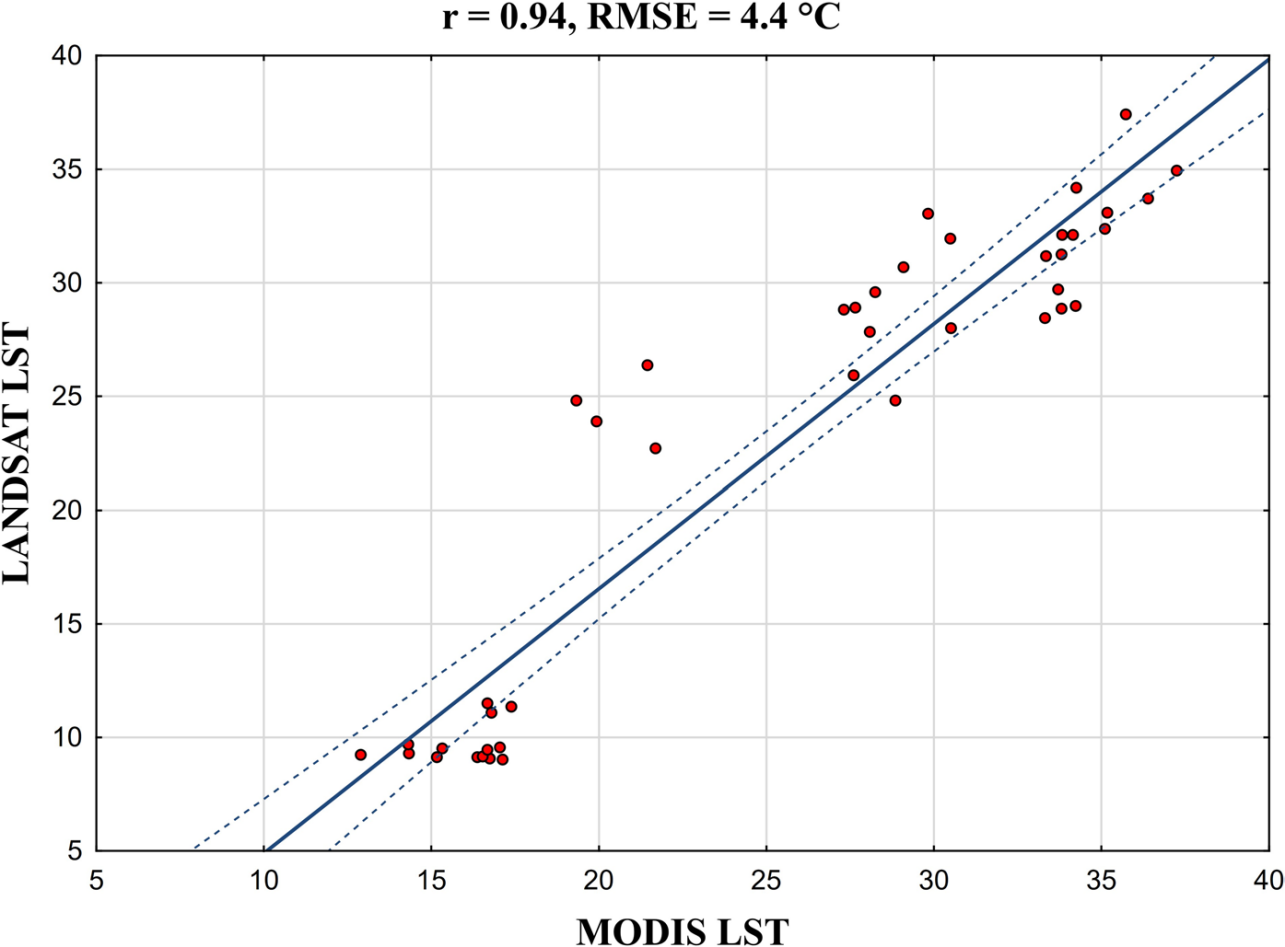
Accuracy validation of Landsat LST with MODIS daily LST

MODIS daily LST data was taken as a reference and compared with Landsat LST for accuracy assessment to test the reliability of the study. Accuracy assessment between two datasets is shown as a scatterplot in Fig. 6, and a strong positive correlation coefficient was obtained between the two datasets as *r* = 0.94, while the overall root mean square error was found to be 4.4 °C.

**Fig. 7:**
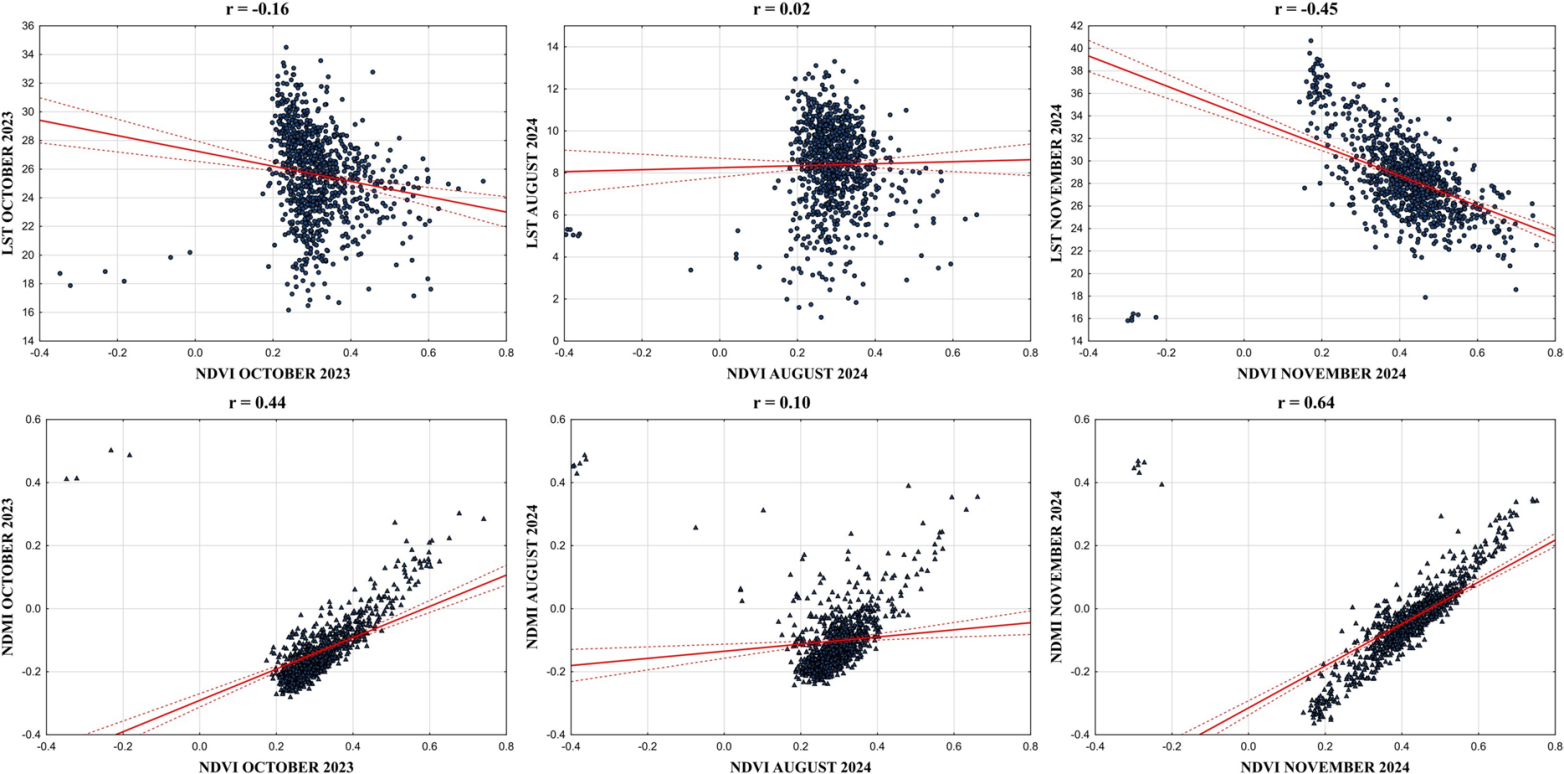
NDVI relationship with LST and NDMI (*p* ≤ 0.05)

The statistical assessments were calculated between spectral RS indices using 927-pixel points for a better understanding of their relationships with each other. According to NDVI relationships with LST and NDMI, which are shown in Fig. 7, LST had a negative correlation coefficient with NDVI as *r* =  − 0.45 after the wildfire in November 2024. The relationship of NDVI with LST was also negative in October 2023 as *r* =  − 0.16, but the weakest correlation coefficient between NDVI and LST was 0.02 in August 2024. In addition, scatterplots between NDVI and LST have more linear distributions in November 2024, while there is high dispersion in the data before the wildfire in October 2023 and August 2024. On the other hand, the pixels created a more dispersed appearance on the scatterplot before the wildfire. Moreover, the NDMI has a positive correlation coefficient with NDVI both in October 2023 and November 2024, but it was the strongest positive correlation coefficient in November 2024 as *r* = 0.64. The lowest correlation was found between NDMI and NDVI in August 2024 as *r* = 0.10.

**Fig. 8:**
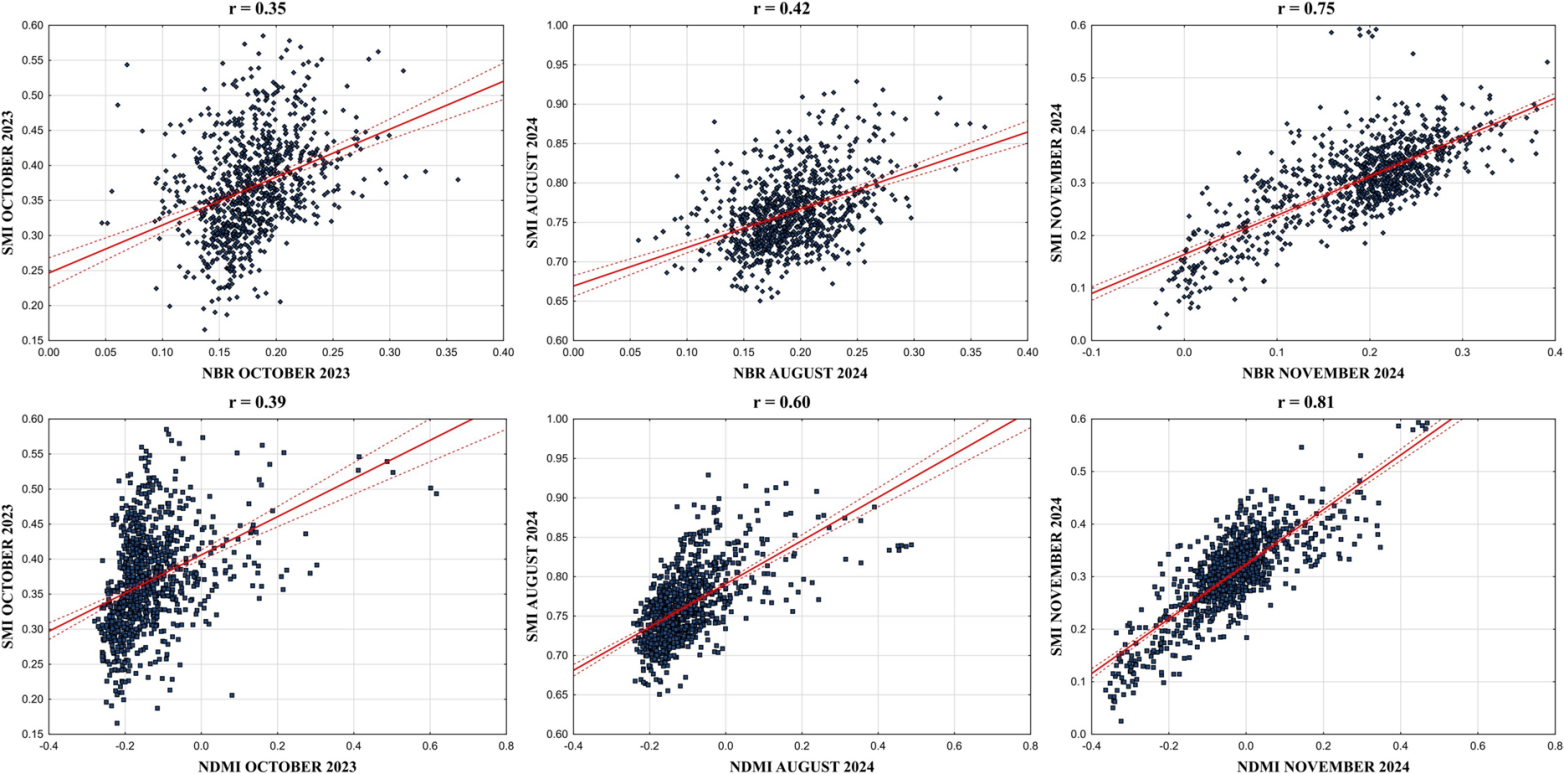
SMI relationship with NBR and NDMI (*p* ≤ 0.05)

The statistical relationships of SMI with NBR and NDMI are shown in Fig. 8 as scatterplots. The SMI has a positive relationship with NBR for all time, but there was high dispersion of pixels on the scatterplots in October 2023 and August 2024 before the wildfire. The strongest positive relationship between SMI and NBR was *r* = 0.75 in November 2024 after the wildfire, while the lowest correlation coefficient was *r* = 0.35 in October 2023 before the wildfire. Moreover, the NDMI relationship with SMI was found with strong positive correlation coefficients for all time, and the relationships have an increasing trend from October 2023 to November 2024. The highest NDMI and SMI correlation coefficient was found (*r* = 0.81) in November 2024, whereas the lowest relationship was *r* = 0.39 in October 2023. Additionally, the results showed that the relationship between SMI and NDMI was stronger than its relationship with NBR. According to statistical validation, it is observed that the relationships between the RS indices became stronger after the wildfire, and data are more dispersed on the scatterplots in October 2023 and August 2024 before the wildfire, while it is more linear and reliable in November 2024 after the wildfire.

**Fig. 9:**
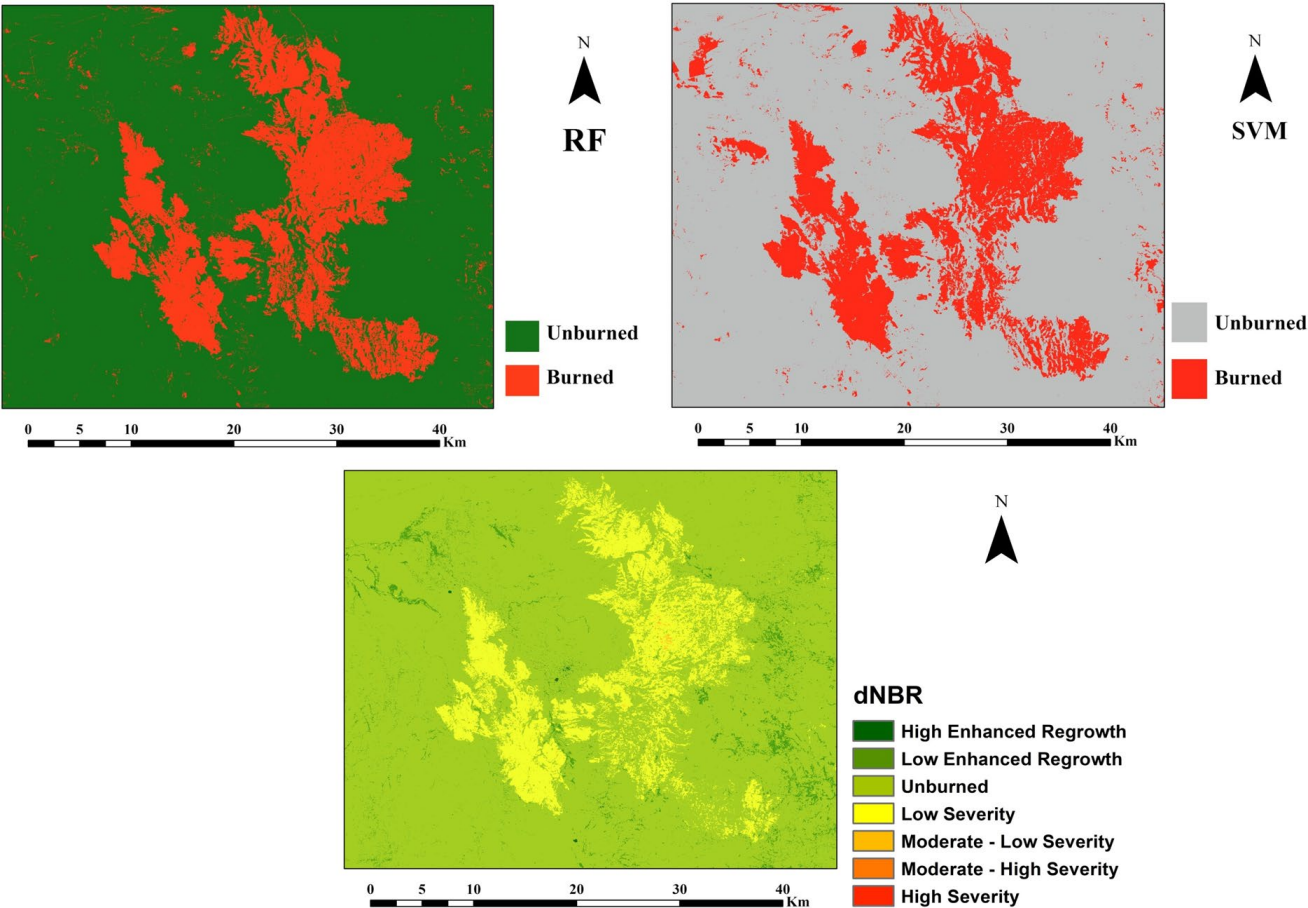
dNBR, random forest (RF), and support vector machine (SVM) classification maps for estimation of burned areas in the study area.

Burned areas are estimated using supervised ML classification methods, which are RF and SVM, and the estimation of burned areas was calculated as 392 km^2^ by RF, while it was 389.5 km^2^ by SVM. In addition to the ML classification, burned areas were also calculated as 387.9 km^2^ using dNBR. Supervised ML classification results of unburned and burned areas and the fire severity map of dNBR are shown in Fig. 9. According to dNBR, low severity was observed in burned areas. dNBR values of 0.10 and above indicate fire severity, and values higher than 0.66 indicate high severity. Red areas represent burned areas in both ML supervised classification maps, and the results showed that there is a similar distribution of burned pixels in the study area. In addition, the overall accuracy of SVM and RF classifications is 97% and 100%, respectively, while kappa coefficients are 0.94 for SVM and 1.0 for RF. Accuracy assessment of RF and SVM was done using validation datasets, which were selected as 30% of 1400-pixel points.

**Fig. 10:**
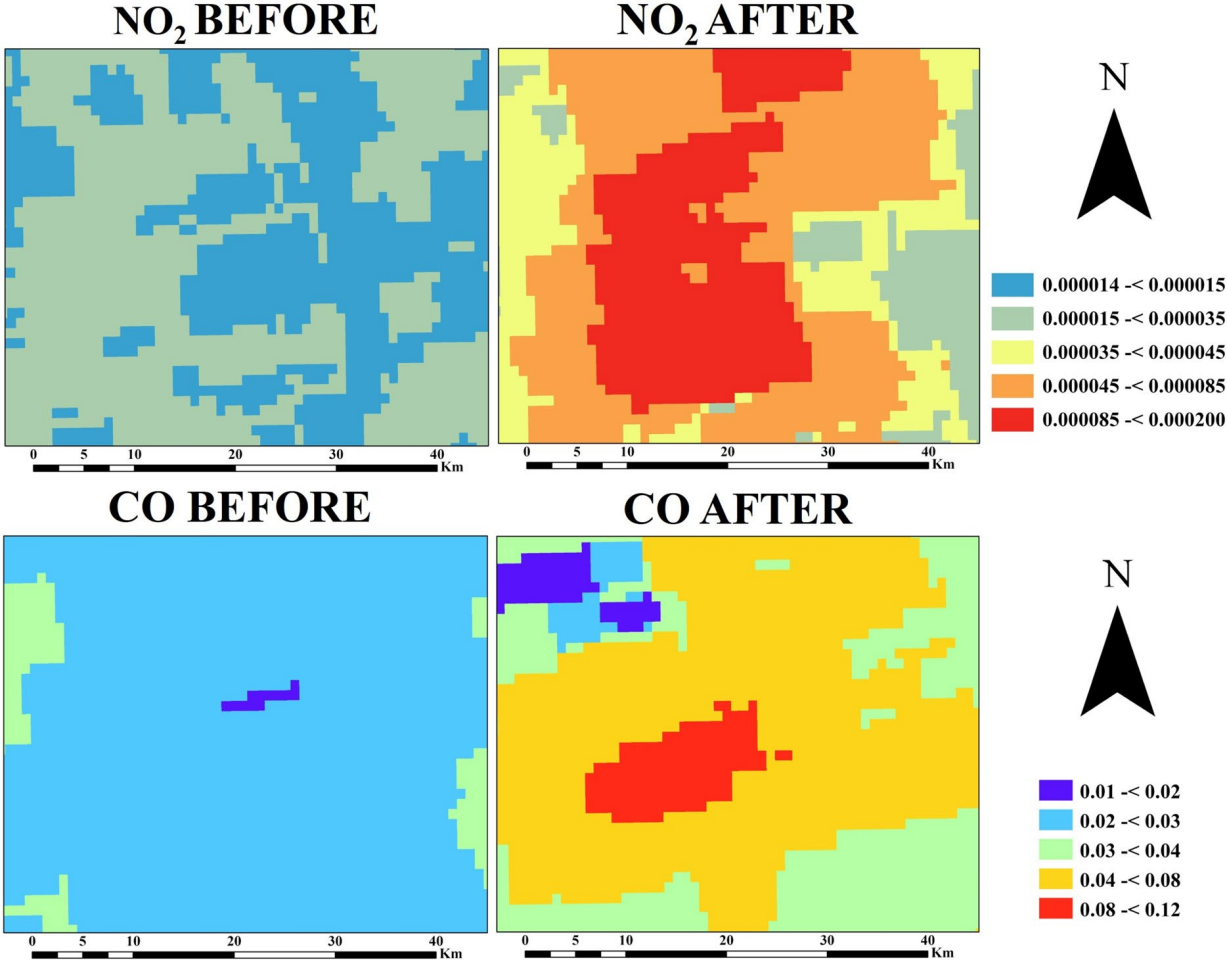
Nitrogen dioxide (NO_2_) and carbon monoxide (CO) distribution (mol/m^2^) for before and after the wildfire in the study area

The changes of air quality before and after the wildfire are shown in Fig. 10, which demonstrated that there are significant changes in NO_2_ and CO concentrations after the wildfire. The NO_2_ concentration was found to be lower than 35 × 10^−5^ (mol/m^2^) before the wildfire, while it was higher than 35 × 10^−5^ (mol/m^2^) after the wildfire. In addition, it was observed that the CO concentration before the wildfire was in a range of 0.01 and 0.04 (mol/m^2^), while after the wildfire, it was between 0.03 and 0.12 (mol/m^2^).

## Discussion

The impacts of a wildfire in Capilla del Monte, located in the Punilla Valley, were investigated using Landsat-9 RS data on the cloud-based GEE platform. Changes in vegetation cover, soil surface temperature, and moisture of plant and soil were observed by comparing spectral RS indices such as NDVI, NBR, NDMI, LST, and SMI before and after the wildfire. Soil moisture and vegetation cover are important to understand the soil health and biodiversity loss after wildfires. NDVI, NDMI, LST, and SMI demonstrate seasonal dynamics due to changes in vegetation greenness, phenology, and climatic conditions throughout the year. NDVI generally peaks during the growing season and declines during dry or calm seasons when there are no disturbances such as wildfires. Thus, photosynthetic activity is lower in August 2024 due to the winter season, while October 2023 and November 2024 represent increased vegetation greenness because of spring regeneration. It is important to monitor different seasonal variability by comparing post-fire results in November 2024 to both the pre-fire spring season and winter season to understand that declines in post-fire vegetation indices are not solely due to natural phenological cycles but are directly linked to fire damage. In addition, the NDVI map in November 2024 had more green pixels compared to August 2024 and October 2023, providing a better opportunity to observe burned pixels. In addition, a comparison of NDMI maps from November 2024 and October 2023 shows that red and lower NDMI values after the wildfire typically indicate drier conditions, which are inevitable following fire events and can lead to increased fire susceptibility (Gupta & Shukla, 2022).

According to Table 1, the mean NDVI increased from 0.32 to 0.43 when comparing data from October 2023 to November 2024. However, it is observed that the SMI decreased by approximately 16% during the same period. Although this represents the same seasonal period, the increase in surface temperature due to the forest fire may have triggered increased evaporation, which can be considered a reason for the decrease in SMI. While NDVI values suggest a visual recovery of vegetation, the simultaneous reduction in SMI indicates that soil moisture conditions remain stressed. This suggests a delayed eco-hydrological recovery, where surface greening does not fully reflect the ecosystem’s functional resilience following the wildfire. From a biological perspective, the rapid increase in NDVI may be primarily driven by early successional or fire-adapted species capable of quickly colonizing disturbed areas. In contrast, species that depend on deeper root systems or stable soil moisture may remain limited due to post-fire reductions in water availability. Apart from this, the positive correlation coefficients between NDVI and NDMI, with the strongest correlation observed in November 2024 as *r* = 0.64, indicate that plant moisture content increased as vegetation recovered (Zhang et al., 2023). This situation may also be due to rainfall in the valley after the wildfire, as well as the seasonal change from winter to spring (Elena et al., 2021). In addition to this, Hope et al. (2012) have highlighted that moisture availability directly influences the rate and extent of vegetation regrowth in wildfire-affected ecosystems, as it supports plant establishment and soil stabilization.

As expected in post-fire ecosystems, the negative relationship between NDVI and LST indicates cooler surface temperatures in areas with increased vegetation cover. Similarly, a negative correlation between LST and NDVI was observed in the Mediterranean forests of Morocco by Ezzaher et al. (2023) following wildfire. Moreover, in the Tirupati region, a significant post-fire change in NDVI and LST was observed, with a 35.8% increase in NDVI and a 14.69% decrease in mean surface temperature (Digavinti and Manikiam, 2021). Although the LST maps from October 2023 and November 2024 both represent the spring season, LST was higher in November 2024 due to the wildfire. The mean LST values presented in Table 1 also support this observation, as November 2024 is 2.4 °C warmer than October 2023, despite only a 16-day gap between the two satellite images. In addition, the reason for the lowest LST values observed in August 2024 can be related to the winter season. Furthermore, the accuracy of LST estimation was assessed by comparing it with MODIS daily 1 km LST reference satellite data, as conducted by Dursun et al. (2025), and their study found a similarly strong positive correlation between the two datasets.

Positive correlation coefficients between SMI and both NBR and NDMI indicate the reliability of moisture dynamics between soil and vegetation. The highest correlation between SMI and NBR (*r* = 0.75) in November 2024, when burned areas were most prominent, indicates that decreases in soil moisture are closely aligned with the spectral signals of burn severity. The increase in correlation coefficients observed after wildfire, along with the more clustered linear distributions in the scatter plots, may be a result of the fire reducing surface heterogeneity by removing various vegetation types, leaving a more uniform surface dominated by bare soil and ash. Removal of vegetation cover reduces the complexity of surface temperature dynamics and increases the correlation between indices such as SMI and NDMI by exposing soil moisture to direct atmospheric influence.

According to the wildfire report published by authorized units on 26th September 2024, toxic gases were released into the environment during the wildfire, adversely affecting the health of residents. The report also stated that the ecosystem and the environment suffered great damage with an estimated area of approximately 430 km^2^ burned in the Punilla Valley and Calamuchita regions (Defensoría del Pueblo de la Nación, 2024). In addition, burned areas in Capilla del Monte were estimated using 10 m spatial resolution Sentinel-2 satellite data to be approximately 420.5 km^2^ by IDECOR (Infraestructura de Datos Espaciales de la Provincia de Córdoba) (https://www.idecor.gob.ar/incendios-en-el-tercer-trimestre-como-afectaron-a-los-bosques-nativos/), while CONAE (Comisión Nacional de Actividades Espaciales) (https://www.argentina.gob.ar/noticias/monitoreo-satelital-de-los-incendios-en-cordoba-y-san-luis) reported a similar estimate of 420.3 km^2^ using Sentinel-2 remote sensing satellite images that were taken on 03rd October 2024. Since the Calamuchita region is not included in our study, the burned area within our study covering the Punilla Valley was estimated to be 387.9 km^2^ using the dNBR method, with burned areas covering approximately 19.8% of the total study area. In addition, the burned areas were calculated using RF and SVM ML methods, estimating 392.4 km^2^ with RF and 389.5 km^2^ with SVM. A discrepancy of less than 40 km^2^, approximately 10% of the total burned area (~ 390 km^2^), suggests that both the RF and SVM methods are highly consistent and robust in capturing the post-fire spectral signal. This minor difference indicates that uncertainties related to training data, parameter tuning, and sensor noise are minimal. Therefore, the results demonstrate that, despite their different algorithmic foundations, both classifiers effectively distinguish between burned and unburned areas. The dNBR was also calculated to determine the fire severity, as in the study conducted by Çolak and Sunar (2023). Moreover, prior studies have shown that while SVM demonstrates high accuracy, the relative performance of RF and SVM varies depending on the context (Chandel et al., 2022; Chen et al., 2024; Mashhadi & Alganci, 2021; Xie and Shi, 2014). For example, Tan et al. (2024) found that combining RF with object-based methods yielded the highest accuracy for detecting burn areas and vegetation recovery after the 2022 wildfire in Portugal. Similarly, Roteta and Oliva (2020) optimized an RF classifier to accurately detect burned areas across diverse biomes in Chile, outperforming official burn perimeter data. Additionally, Ramo and Chuvieco (2017) reported that RF models achieved balanced accuracies of up to 0.94 for global burned area detection using MODIS imagery. These results, consistent with the current study, indicate that both RF and SVM are robust tools for capturing post-fire spectral signals, with minimal uncertainties from training data, parameter tuning, and sensor noise.

The wildfire significantly affected air quality in the Punilla Valley, with approximately two-fold increases observed in the concentrations of some toxic gases, such as NO_2_ and CO, after the wildfire. NO_2_ levels exceeding 35 × 10⁻^5^ mol/m^2^ and CO concentrations exceeding 0.12 mol/m^2^ highlight the fire’s contribution to atmospheric degradation, posing potential health risks and ecological challenges in the region. A similar trend was observed in the study conducted by Yilmaz et al. (2023) using Sentinel-5P data for 2021 wildfires in Turkey, and it was seen that the average CO level during the fire increased from 0.03 to 0.14 mol/m^2^. Since increased concentrations of toxic gases result from the combustion of organic matter in the soil, the intensity of these gas concentrations can provide an estimate of the amount of organic matter burned. The burning of organic matter in the soil causes soil degradation, and the resulting ash blocks soil pores, thereby reducing water infiltration. Since rainwater cannot infiltrate the soil, runoff increases. Although the decrease in soil moisture after the fire is partly due to evaporation, it may also be influenced by the increased runoff rate. The same condition was also stated by Zhou et al. (2025); soil erosion may be triggered due to the deterioration of soil structure because of the burning of organic matter, and an increase in water run-off is expected.

The study’s results provide critical insights for forest management regarding the interrelationships among vegetation, soil moisture, and thermal dynamics in post-wildfire ecosystems. Moreover, recent developments in cloud-based platforms like Google Earth Engine have facilitated the integration of RF and SVM classifiers, providing a scalable framework to apply this approach in other regions (Nelson et al., 2024). Despite the study’s benefits, several limitations affect the reliability of the results. The lack of higher temporal resolution in satellite imagery restricts the ability to observe vegetation recovery and short-term fluctuations in soil moisture. While calculating SMI using LST minimum and maximum values effectively captures soil moisture changes under typical conditions, extreme temperature differences in wildfire-burned areas may affect the accuracy of SMI. Moreover, considering the high correlation among the RS indices used in the supervised classifications, and the SMI is derived from LST data, there is a potential risk of overfitting, which may affect the supervised classification accuracy. Additionally, the presence of aerosols and particulate matter in the atmosphere during satellite data acquisition can affect atmospheric transmittance, leading to uncertainties in spectral index calculations. These limitations align with challenges reported in SVM-based studies of post-fire NDVI recovery, where coarse-resolution data obscured short-term vegetation resilience (Kurbanov et al., 2022).

## Conclusion

The wildfire that occurred in Capilla del Monte, one of Argentina’s major tourist regions, was analyzed using RS indices. In the study, the estimation of burned areas was successfully conducted using the NBR index, and the dNBR analysis determined that approximately 387.9 km^2^ of land was burned in the region, while the burned area was estimated at 392 km^2^ using the RF ML classification and 389.5 km^2^ using the SVM. In areas with dense vegetation, surface temperatures decrease due to the cooling effect, whereas in sparsely vegetated or bare areas, surface temperatures tend to increase. The relationship between SMI and NBR is not commonly used in wildfire studies; however, it demonstrated a positive trend, with greater data dispersion observed before the wildfire. In addition, it was observed that the concentrations of NO_2_ and CO gases released after the fire increased to approximately twice or more their pre-fire levels. In summary, the harmful gases released due to the wildfire posed a serious threat to the health of the regional ecosystem, and an increase in surface temperature and a decrease in soil moisture were observed following the wildfire. This observation may negatively affect plant growth, especially when considering the temperature increases expected during the summer months. In conclusion, this study highlights the effectiveness of RS indices in assessing the impacts of wildfires on vegetation and soil moisture. Future studies should incorporate observations at more frequent intervals using higher temporal resolution data and achieve greater accuracy by correlating remote sensing results with ground-truth data.

## Data Availability

No datasets were generated or analysed during the current study.
